# P-1277. Impact of Common Biases on Simulated Relative Vaccine Effectiveness Estimates: Implications for Evidence Synthesis, Decision Making, and Real-World Studies

**DOI:** 10.1093/ofid/ofae631.1458

**Published:** 2025-01-29

**Authors:** Daniel Harris, Matthew M Loiacono, Ayman Chit, Arman Oganisian, Robertus van Aalst

**Affiliations:** Department of Epidemiology, College of Health Sciences, University of Delaware, Newark, Delaware; Sanofi, Swiftwater, Pennsylvania; Sanofi Pasteur, Swiftwater, PA; Department of Biostatistics, Brown University, School of Public Health, Providence, RI, USA, Providence, Rhode Island; Sanofi Vaccines, Lyon, Auvergne, France

## Abstract

**Background:**

A growing number of real-world studies are using health administrative data to estimate relative vaccine effectiveness (rVE) due to ease of access and lower cost. To inform evidence-based decision making, meta-analyses of rVE estimates are often employed to summarize the volume of studies; however, low statistical power, unmeasured confounding, and publication bias threaten the validity of meta-analyzed estimates. Our objective was to demonstrate the impact of these biases on meta-analyzed rVE estimates using simulation.

**Methods:**

We simulated a binary, enhanced vs. standard influenza vaccination strategy (allocated ∼1:1) and rare binary outcome (< 1.0%). True rVE was 20% (1–risk ratio [RR]=0.80). To simulate observational studies, weak negative confounding due to age (RR=1.02) and strong negative confounding by indication (RR=2.0) were introduced as measured and “unmeasured” confounders, respectively. Generalized linear models (link=log; distribution=binomial) estimated RRs and 95% profile confidence intervals (CI). To assess statistical power, small (N=5,000-10,000) and large (N=100,000-150,000) “studies” were conducted. Meta-analysis pooled rVEs by study design and size (randomized [30 small; 30 large]; observational [30 small; 30 large]). To emulate publication bias, statistically significant rVEs were pooled separately.

**Results:**

Vaccine effectiveness point estimates varied widely in small samples (e.g., randomized rVE range = 42% to -18%; observational rVE range = 36% to -78%). Large, randomized studies were unbiased and precise on average (pooled rVE=21%; 95%CI=20-23), while large observational studies were biased and precise (pooled rVE = -4%; 95%CI=-2 to -6). Pooled rVE estimates from small and statistically significant randomized studies overestimated true rVE (n=2; pooled rVE=41%; 95%CI=19-57). Pooling all studies underestimated true rVE (n=120; pooled rVE=4%; 95%CI=3-5).

**Conclusion:**

Low statistical power, confounding, and publication biases contributed to biased, pooled rVE estimates in this simulation. Public health decision-makers using meta-analyses should be aware of these threats to validity. Additional guidance to identify/address these potential biases in meta-analysis are needed.

**Disclosures:**

**Daniel Harris, PhD, MPH**, Sanofi Vaccines: Advisor/Consultant **Matthew M. Loiacono, PhD, MSc**, Sanofi: full-time employee|Sanofi: Stocks/Bonds (Public Company) **Ayman Chit, MBiotech, PhD**, Sanofi: I am an employee of Sanofi|Sanofi: Stocks/Bonds (Public Company) **Robertus van Aalst, PhD, MSc**, Sanofi: Employee|Sanofi: Stocks/Bonds (Public Company)

